# Vaccination with viral vectors expressing NP, M1 and chimeric hemagglutinin induces broad protection against influenza virus challenge in mice

**DOI:** 10.1016/j.vaccine.2019.07.095

**Published:** 2019-09-03

**Authors:** Guha Asthagiri Arunkumar, Meagan McMahon, Vincent Pavot, Mario Aramouni, Andriani Ioannou, Teresa Lambe, Sarah Gilbert, Florian Krammer

**Affiliations:** a Department of Microbiology, Icahn School of Medicine at Mount Sinai, New York, USA; b Graduate School of Biomedical Sciences, Icahn School of Medicine at Mount Sinai, New York, USA; c The Jenner Institute, University of Oxford, Oxford, UK

**Keywords:** Influenza, Stalk, NP, M1, T-cell immunity, Universal influenza virus vaccine

## Abstract

Seasonal influenza virus infections cause significant morbidity and mortality every year. Annual influenza virus vaccines are effective but only when well matched with circulating strains. Therefore, there is an urgent need for better vaccines that induce broad protection against drifted seasonal and emerging pandemic influenza viruses. One approach to design such vaccines is based on targeting conserved regions of the influenza virus hemagglutinin. Sequential vaccination with chimeric hemagglutinin constructs can refocus antibody responses towards the conserved immunosubdominant stalk domain of the hemagglutinin, rather than the variable immunodominant head. A complementary approach for a universal influenza A virus vaccine is to induce T-cell responses to conserved internal influenza virus antigens. For this purpose, replication deficient recombinant viral vectors based on Chimpanzee Adenovirus Oxford 1 and Modified Vaccinia Ankara virus are used to express the viral nucleoprotein and the matrix protein 1. In this study, we combined these two strategies and evaluated the efficacy of viral vectors expressing both chimeric hemagglutinin and nucleoprotein plus matrix protein 1 in a mouse model against challenge with group 2 influenza viruses including H3N2, H7N9 and H10N8. We found that vectored vaccines expressing both sets of antigens provided enhanced protection against H3N2 virus challenge when compared to vaccination with viral vectors expressing only one set of antigens. Vaccine induced antibody responses against divergent group 2 hemagglutinins, nucleoprotein and matrix protein 1 as well as robust T-cell responses to the nucleoprotein and matrix protein 1 were detected. Of note, it was observed that while antibodies to the H3 stalk were already boosted to high levels after two vaccinations with chimeric hemagglutinins (cHAs), three exposures were required to induce strong reactivity across subtypes. Overall, these results show that a combinations of different universal influenza virus vaccine strategies can induce broad antibody and T-cell responses and can provide increased protection against influenza.

## 1 Introduction

The effectiveness of annual vaccination in reducing the morbidity and mortality caused by seasonal influenza virus infection strongly depends on the extent to which the selected vaccine strains match the circulating viruses. The protection conferred is restricted to seasonal influenza virus, and does not cover emerging pandemic influenza virus strains. Current seasonal influenza virus vaccines induce antibody responses against the hemagglutinin (HA), the major surface glycoprotein of the virus [1]. The majority of the antibodies induced by these vaccines target the variable, immunodominant HA head domain and are capable of neutralizing the virus by preventing receptor interaction and viral entry. However, most of the antigenic drift is mediated by the very plastic globular head domain making annual re-formulation and re-administration of the vaccine necessary. Mismatches between vaccine strains and circulating strains frequently occur, leading to a sharp decline in vaccine effectiveness [2], [3]. Recent approaches to develop a universal influenza virus vaccine involve refocusing the immune response away from the globular head domain, towards the immunosubdominant, but conserved stalk region of the HA or to internal proteins like the nucleoprotein or the matrix protein 1 which are also highly conserved [4], [5].

Chimeric hemagglutinin constructs (cHA) are comprised of a stalk domain derived from seasonal H1, H3 or influenza B virus HAs and HA head domains from avian influenza virus subtypes to which the general population is immunologically naïve to [6]. Sequential vaccination with cHAs that have the same stalk but very different head domains can refocus the immune response to the stalk. This is achieved because repeated exposure to the same immunogen (the stalk) leads to strong recall responses while only primary responses are mounted to the drastically different head domains. Although these avian head domains are immunogenic, the overall response is predominantly against the seasonal HA stalk domains given that humans have not encountered these avian viruses in circulation previously and are therefore not primed. This strategy has been successfully tested in animal models using traditional vaccine platforms (recombinant proteins, split vaccine, live attenuated vaccines) [7], [8], [9], [10], [11], [12], [13], [14] but also worked reasonably well when viral vectors were used [15], [16], [17]. So far, the cHA concept has been tested widely for group 1 HAs but relatively little work has been performed with group 2 HAs. The majority of isolated human anti-stalk antibodies react to either group 1 (H1, H2, H5, H6, H8, H9, H11, H12, H13, H16, H17 and H18) or group 2 (H3, H4, H7, H10, H14, H15) HAs but do not cross-react between groups; suggesting that both a group 1 and a group 2 component for a universal influenza virus vaccine will be required. Therefore, and because group 2 viruses like H3N2 cause high morbidity and mortality in humans, work on a group 2 vaccine component is important. While some universal influenza virus vaccine candidates based on the stalk domain of HA aim at inducing antibody responses, other strategies focus on inducing T-cell responses to internal proteins such as nucleoprotein (NP) and the matrix protein 1 (M1) which are highly conserved across seasonal and zoonotic influenza viruses [5]. It has been shown that T-cell responses targeting these epitopes can be highly protective in the mouse model [18], [19], [20] and both CD8+ and CD4+ T-cells correlate with protection in humans, also in elderly populations [21], [22], [23], [24], [25]. To optimally induce targeted T-cell responses, NP and M1 need to be expressed intracellularly, e.g. using replication deficient viral vectors like Chimpanzee Adenovirus Oxford 1 (ChAdOx1) and Modified Vaccinia Ankara (MVA) [26], [27], [28], [29], [30], [31], [32], [33]. In addition to these viral vectors expressing NP and M1, they have also been supplemented with or given the capacity to express H5 and H7 full length HA. These vectors induced strong humoral and cellular responses against all three antigens. The induced immune responses are augmented by the naturally adjuvanting nature of these viral vectors [34], [35].

In this study, we combined these two concepts using ChAdOx1 and MVA vectors expressing group 2 cHAs and NP+M1 from the same viral vector and determined the protective effect against group 2 HA expressing viruses in a mouse model. Challenge viruses included H3N2, H7N9 and H10N8. Overall, our findings suggest that combining the two different vaccine approaches leads to enhanced protection *in vivo*.

## 2 Materials and methods

### 2.1 Cells and viruses

Sf9 cells for baculovirus rescue were grown in *Trichoplusia ni* medium-formulation Hink (TNM-FH) insect cell medium (Gemini Bioproducts) supplemented with 10% fetal bovine serum (FBS) and penicillin-streptomycin (100 U/ml penicillin, 100 μg/ml streptomycin) solution (Gibco). BTI-TN-5B1-4 (High Five) cells for protein expression were grown in serum-free SFX medium (HyClone) supplemented with penicillin-streptomycin solution. Madin Darby Canine Kidney (MDCK) cells were grown in Dulbecco’s Modified Eagle’s Medium (DMEM) supplemented with 5% FBS, penicillin-streptomycin solution and 5% 1 M 4-(2-hydroxyethyl)-1-piperazineethanesulfonic acid (HEPES) solution (Gibco). The influenza A virus strains A/Philippines/2/82 (H3N2, X-79), A/Shanghai/1/13 (H7N9) and A/Jiangxi-Donghu/346/13 (H10N8) were all reassortants carrying the internal genes from vaccine strain A/PR/8/34 (PR8; H1N1) and were grown in 10-day-old embryonated chicken eggs (Charles River) for 48 h at 37 °C. Eggs were then cooled overnight at 4 °C before harvesting the allantoic fluid. The allantoic fluid was centrifuged at 2000 g for 10 min at 4 °C to remove debris. Viruses were aliquoted and stored at −80 °C. For virus purification, viruses were grown as described above (including the low-speed centrifugation step). Virus was then pelleted by centrifugation at 25,000 rpm using a SW-28 rotor in a Beckman L7-65 ultracentrifugre for 2 h at 4 °C in 1 × NTE buffer (0.5 mM NaCl, 10 mM Tris-HCl, pH 7.5, 5 mM EDTA) over a 30% sucrose cushion.

### 2.2 Recombinant proteins

Soluble A/Philippines/2/82 H3, A/Shanghai/1/13 H7 and A/Jiangxi-Donghu/346/13 H10 HA containing a T4 foldon trimerization domain and a C-terminal hexahistidine tag for purification were generated using the baculovirus protein expression system as previously described [36], [37]. NP and M1 from A/PR/8/34 with N-terminal hexahistidine tags were expressed using the baculovirus system as well and were purified from Sf9 cell lysates. Additionally, A/Perth/16/09 H3 HA was expressed with a GCN4pII trimerization domain and a C-terminal streptavidin-tag for purification to avoid a background signal in enzyme linked immunosorbent assay (ELISA) caused by anti-histidine or anti-trimerization domain antibodies.

### 2.3 Viral vectors

The E1 locus and a CMV promotor were used to drive antigen expression for the ChAdOx1 vector. For monovalent vaccines cH14/3 (head domain from A/mallard/Gurjev/263/1982 (H14N5) plus stalk domain from A/Perth/16/09 (H3N2)) [9] or NP+M1 (from H3N2 strain A/Panama/2007/1999, joined by a 7 amino acid linker) were expressed [30]. For bivalent vaccines a 2A ribosome skipping site was inserted between NP+M1 and cH14/3. Expression from the MVA vectors [38] was driven using an endogenous F11 promoter [39] for NP+M1 and the mH5 promotor [40] for cH15/3 (head domain from A/shearwater/Western Australia/2576/1979 (H15N9) plus stalk domain from A/Perth/16/2009 (H3N2)) [9] at the F11 site. The bivalent expression construct was made by inserting the NP-M1 under F11 control followed immediately by the cH15/3 under mH5 control within the F11 insertion site.

The vectors were produced by the Jenner Institute’s Viral Vector Core Facility in T-Rex-293 cells (for ChadOx1, ThermoFisher) or DF-1 cells (MVA) [41] and purified via sucrose cushion centrifugation.

### 2.4 Animal experiments

Six to eight week old female BALB/c mice (Jackson Laboratories) were used for all animal experiments. All experiments were performed in accordance with protocols approved by the Icahn School of Medicine at Mount Sinai Institutional Animal Care and Use Committee (IACUC).

**Fig. 1:**
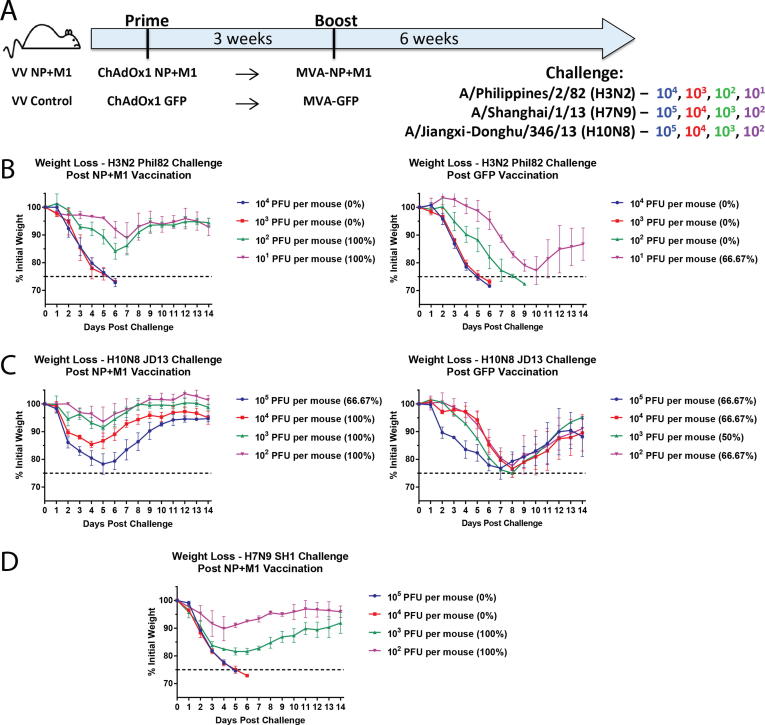
Determination of the optimal challenge dose of group 2 influenza viruses. (A) Mice were primed IM with ChAdOx1 NP+M1 and boosted with MVA-NP+M1 three weeks later. (ChAdOx1 then MVA). Six weeks post-boost, mice were challenged intranasally with 10-fold increasing doses of A/Philippines/1982 (H3N2, X-79), A/Jiangxi-Donghu/346/2013 (H10N8, PR8 reassortant) and A/Shanghai/1/2013 (H7N9, PR8 re-assortant). For H3N2 and H10N8 control mice vaccinated with the same virus vectors but expressing GFP were added. (B), (C), & (D) Weight loss plots for vaccinated mice challenged with H3N2, H7N9, and H10N8 respectively are shown over a 14 day time period. Weight loss is represented as mean of the group with error bars representing SD. *n* = 3 mice/dose/challenge group. The percentage values indicated in the parenthesis refer to the survival percentage in each group.

To determine an optimal challenge dose of group 2 influenza viruses in vaccinated mice, the mice were divided into 2 groups (3 mice per group per challenge virus dose) (Fig. 1A). In VV NP+M1, mice received a prime and a boost of ChAdOx1 NP+M1 and MVA-NP+M1, respectively, 3 weeks apart. Mice in the VV Control group received the corresponding ChAdOx1 and MVA green fluorescent protein (GFP) control vectors 3 weeks apart. The vaccinations were administered intramuscularly (IM) at 1x10^8^ infectious units (IU)/50 µL in phosphate buffered saline (PBS) for the chimpanzee adenovirus vectors, and 1x10^6^ plaque forming units (pfu)/50 µL in PBS for the MVA vectors. Both groups were then challenged intranasaly (IN) with log incremental doses of A/Philippines/2/82 (H3N2, X-79), A/Shanghai/1/13 (H7N9) or A/Jiangxi-Donghu/346/13 (H10N8) (all 6:2 A/PR/8/34 reassortants). The survival and weight loss was monitored over 14 days, with a 25% weight loss cut-off used as a humane end point following which mice were euthanized. For H7N9, only the ChAdOx1 NP+M1 and MVA-NP+M1 group was tested. As stated above, three mice per vaccination group per challenge dose were used. The exception was the 10^3^ PFU per mouse group for A/Jiangxi-Donghu/346/13 (H10N8) which included only two mice in that group. One mouse in this group was pre-emptively sacrificed prior to the 25% weight loss cut off and was excluded from the study.

**Fig. 2:**
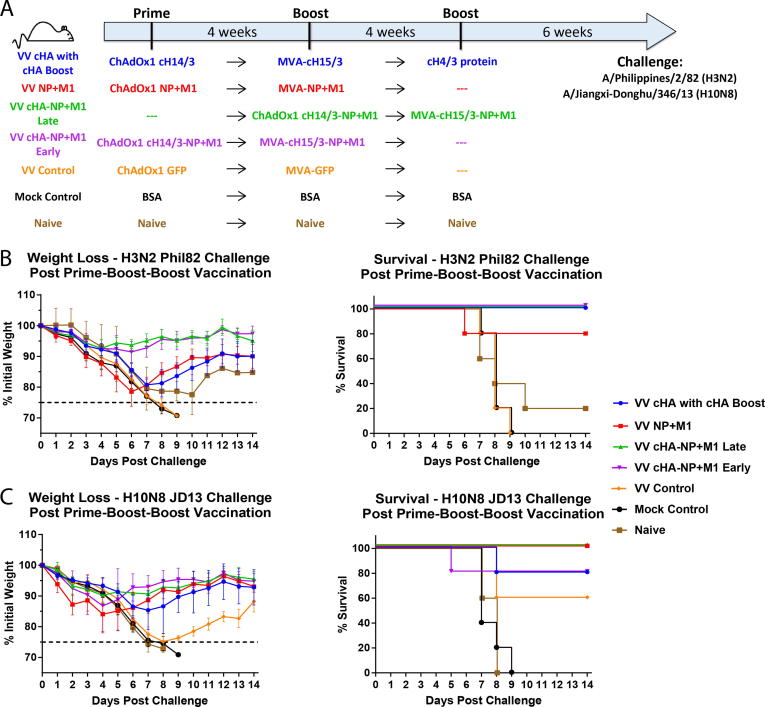
Assessing the protective effect of viral vectored vaccines against challenge with H3N2 and H10N8 viruses. (A) Mice were vaccinated intramuscularly as shown in a prime-boost or a prime-boost-boost regimen with four weeks between each vaccination. Groups VV cHA with cHA Boost, VV NP+M1, VV cHA-NP+M1 Early, VV cHA-NP+M1 Late, VV Control and Mock Control received vectored cHAs, vectored NP+M1, vectored cHA-NP+M1 (Early & Late), vectored GFP control and irrelevant protein vaccinations respectively. Six weeks after the boost, mice were challenged intranasally with 10^2^ pfu/mouse of A/Philippines/2/82 (H3N2, X-79) or 10^4^ pfu/mouse A/Jiangxi-Donghu/346/13 (H10N8, PR8 reassortant). (B) and (C) Weight loss and Kaplan-Meier survival plots are shown over 14 days post challenge with A/Philippines/2/82 (H3N2), and A/Jiangxi-Donghu/346/13 (H10N8). Weight loss is shown as mean of the group with error bars representing SD. *n* = 5 mice/group.

For experiments to assess the protective effects of monovalent (cHA or NP+M1) and bivalent (cHA-NP+M1) viral vectors against influenza virus infection, the mice were divided in to 7 experimental groups (Fig. 2A). In the VV cHA with cHA group, mice were primed intramuscularly (IM) with 1 × 10^8^ infectious units (IU) of ChAdOx1-cH14/3 in 50 µL of PBS, four weeks later they were boosted IM with 1x10^6^ pfu of MVA-cH15/3 in 50 µL of PBS. Four weeks after the initial boost, these mice were boosted IM with 5 µg of recombinant cH4/3 protein adjuvanted with 5 μg of polyI:C (Sigma-Aldrich) in 50 µL of PBS. The VV NP+M1 experimental group was primed IM with 1x10^8^ IU of ChAdOx1 NP+M1 in 50 µL of PBS, four weeks later the animals were boosted IM with 1x10^6^ pfu of MVA-NP+M1 in 50 µL of PBS. VV cHA-NP+M1 Late and VV cHA-NP+M1 Early were primed IM with 1 × 10^8^ IU of ChAdOx1-cH14/3-NP+M1 in 50 µL of PBS, four weeks later they were boosted IM with 1 × 10^6^ pfu of MVA-cH14/3-NP+M1 in 50 µL of PBS. These groups differed by the commencement of the prime vaccination – the Early group was primed four weeks earlier than the third group. The VV Control group was primed IM with 1 × 10^8^ IU of ChAdOx1-GFP in 50 µL of PBS, four weeks later animals in this group were boosted IM with 1 × 10^6^ pfu of MVA-GFP in 50 µL of PBS. For the H7N9 experiment, an additional group was added to bridge to a previously published study. This group received a cH4/3 DNA prime, a cH5/3 protein boost and a final full length H3 protein boost [11]. Mock Control group mice were primed IM with 5 µg of bovine serum albumin (BSA) adjuvanted with 5 µg polyI:C. At 4 and 8 weeks after the prime, mice in the Mock Control group were boosted IM with 5 µg of BSA adjuvanted with 5 µg polyI:C. Naïve group mice were naïve controls and received no vaccination at any time point. Six weeks after the final boost, mice were intranasally (IN) infected with a lethal dose of A/Philippines/2/82 (H3N2), A/Shanghai/1/13 (H7N9) or A/Jiangxi-Donghu/346/13 (H10N8) (all 6:2 A/PR/8/34 reassortants) and weight loss was measured for a 14-day period. A weight loss of more than 25% was considered as a humane endpoint and animals that reached this endpoint were euthanized. The H7N9 challenge was conducted in two independent experiments with the VV cHA with cHA, VV cHA-NP+M1 Early and Naïve groups being present in both.

### 2.5 Enzyme linked immunosorbent assay (ELISA)

ELISA plates (Immulon 4HBX; Thermo Scientific) were coated with 2 µg/mL of recombinant protein or 5 µg/mL of purified virus (50 µL per well) in coating buffer (KPL coating solution, Sera Care) at 4 °C overnight. The following day, the plates were washed three times with PBS containing 0.1% Tween-20 (PBS-T) and blocked in blocking solution (3% goat serum (Gibco), 0.5% non-fat milk in PBS-T) for 1 h at room temperature (RT). After blocking, pre-diluted serum was added to the first well to a final concentration of 1:100 in blocking solution. Serum was then serial diluted 1:3 in blocking solution and incubated at RT for two hours. Plates were then washed three times with PBS-T before adding rabbit anti-mouse IgG horseradish peroxidase conjugated antibody (Rockland) in blocking solution for one hour at RT. Following the secondary antibody incubation, the plates were washed four times with PBS-T to remove residual antibody and the O-phenylenediamine dihydrochloride substrate (Sigma-Aldrich) was added. After 10 min of incubation at RT, the reaction was stopped by adding 50 µL of 3 M HCl (Fisher Scientific) to the mixture. The optical density (OD) was measured at 490 nm on a Synergy 4 plate reader (BioTek). A cutoff value of the average of the OD values of blank wells plus three standard deviations was established for each plate and used for calculating the area under the curve (AUC), which was the readout for this assay.

### 2.6 Antibody-dependent cellular cytotoxicity (ADCC) reporter assay

MDCK cells were seeded at a concentration of 2 × 10^5^ cells/well in white polystyrene 96-well plates (Costar Corning). The following day, the cells were washed with PBS and 100 μL of H3N2 A/Philippines/2/82 (6:2 A/PR/8/34 reassortant) at a multiplicity of infection of 1 in 1 × minimum essential medium (MEM) (10% 10 X MEM (Gibco), 1% 200 mM L-glutamine (Gibco), 1.6% of sodium bicarbonate stock solution (Sigma-Aldrich), 1% stock solution HEPES (Gibco) and 1% penicillin-streptomycin was added to each well. The cells and virus were then incubated for 24 h at 37 °C. The following day, pre-challenge mouse serum was serially diluted (with a starting concentration of 1:10) 2-fold in RPMI 1640 medium (Life Technologies) in a separate 96-well plate. The infection medium was removed from MDCK cells and they were washed with PBS. After the PBS wash, 6.25 × 10^4^ FcγRIIa reporter cells (in 25 µL) (Promega), 25 µL of 1XMEM and 25 µL of diluted sera were added to the MDCK cells. After a 6-hour incubation at 37 °C, 75 µL of the Bio-Glo luciferase substrate (Promega) was added to each well. The cells were incubated in the dark for 10 min before measuring luminescence on a Synergy H1 microplate reader (BioTek). The results were analyzed in GraphPad Prism 10, and the AUC values were determined. The cutoff was defined as the average of the values of the blank wells plus 5 times the standard deviation of the blank wells.

### 2.7 Microneutralisation assay

MDCK cells (2 × 10^5^ cells/ml) were seeded at 100 µL/well in 96-well cell culture plates (Costar Corning), and incubated for 12 h at 37 °C. Pre-challenge sera from vaccinated mice were pooled according to their groups. Pooled sera were treated with receptor destroying enzyme (RDE; Denka Seiken) as per manufacturer’s instructions (1:3 ratio). Following incubation at 37 °C for 18 h, RDE was inactivated with 2.5% sodium citrate (3 volumes) and by incubating at 56 °C for 30 min. The solution was reconstituted to a final serum concentration of 10% with PBS. Samples were assayed in triplicates, starting at a dilution of 1:25, and serially diluted 2-fold in infection media (UltraMDCK media (Lonza) with N-tosyl-L-phenylalanine chloromethyl ketone (TPCK) – treated trypsin) to a final volume of 120 µL. Sixty µl of diluted sera was incubated with 100x of a 50% tissue culture infectious dose (TCID_50_)/60 µL of A/Philippines/82 (H3N2, 6:2 A/PR/8/34 reassortant) in a 96-well plate for one hour on a shaker at room temperature. Hundred µl of virus-serum mixture was transferred onto MDCK cells that had been washed with PBS. After one hour incubation at 37 °C, the mixture was removed and cells were washed with PBS. To the original plate containing serum dilutions, 60 µL of infection media was added to reconstitute the volume to 120 µL. Hundred µl of the serum dilutions were transferred onto the cells, and they were incubated at 37 °C for 48 h. A classical hemagglutination assay was used as a readout wherein 50 µL of the supernatant from the cells was incubated at 4 °C for one hour with 50 µL of 0.5% chicken red blood cells (RBCs, Lampire Biological Laboratories) in PBS in a 96-well V-bottomed plate (Corning). Appropriate no serum and no virus controls were used, and the number of hemagglutination positive wells were counted.

### 2.8 Interferon-γ (IFNγ) enzyme linked immunospot (ELISpot) assay

Spleens were aseptically removed from vaccinated mice three weeks after the second IM boost. Spleens were then pushed through a 70 µM filter (Foxx Life Sciences) to create single-cell suspensions in minimum essential medium eagle with alpha modification (alpha MEM) (Sigma-Aldrich) supplemented with 5% FBS and penicillin-streptomycin solution. Splenocytes were resuspended to 2.5 × 10^6^ cells/mL and 50 µL of the cell suspension was added to a 96-well multiscreen filter plate (Merck Millipore). These plates had been previously coated with the IFNγ specific mAb overnight at 4 °C at 5 µg/mL. The immunodominant H-2K^d^ –restricted (BALB/c) NP_147–155_ (TYQRTRALV) epitope was used to measure immunodominant IFNγ responses towards the NP (4). Splenocytes were stimulated at a final concentration of 1 µg/mL. Medium alone was used as a negative control. The plates were incubated for 18 h at 37 °C and then washed 5 times with PBS. After washing, the detection antibody was added, and the plates were left for 2 h at room temperature. The plates were then washed 5 times with PBS and the streptavidin-alkaline phosphatase was added and the plates were incubated for 1 h at room temperature. The plates were washed as above and the IFNγ secreting cells were detected using the alkaline phosphatase conjugate substrate kit (Bio-Rad), as per the manufacturer’s instructions. Results are expressed as spot forming units (SFU) per million splenocytes, calculated by subtracting the mean negative control response from the mean of each response.

### 2.9 Intracellular cytokine staining (ICS) assay

Influenza virus-specific T cell responses were assessed using the ICS assay. Briefly, splenocytes were stimulated in a 96-well round bottom plate (Falcon) with the peptides or peptide arrays (described above) at a final concentration of 1 µg/mL in the presence of Brefeldin-A (BioLegend) at 37 °C. Five hours later splenocytes were permeabilized and stained for the presence of CD8α (53-6.7, BioLegend), IFNγ (XMG1.2, BioLegend), tumor necrosis factor α (TNFα, MP6-XT22, BioLegend) and interleukin 2 (IL2, Jes6-5H4, BioLegend). Data was acquired on a BD-LSRII and analyzed using FlowJo software. ICS results are expressed as % of CD8+ T cells.

### 2.10 Statistics

The data was presented as individual replicates or means with standard deviation (SD); n represents the number of mice per experiment. Statistical differences between three or more groups were determined by Kruskal-Wallis one-way analysis of variance (ANOVA) with Dunn's correction for multiple comparisons. All statistical analyses were performed using GraphPad Prism 7 for Windows. In all cases, probability levels<0.05 (*p < 0.05; **p < 0.01; ***p < 0.001; ****p < 0.0001) were indicative of statistical significance.

## 3 Results

### 3.1 Determination of the optimal construct sequence and immunization route for vaccination

To determine whether viral vectors expressing cHA constructs could induce anti-stalk antibodies, we vaccinated 6 to 8-week-old female BALB/c mice with a number of viral vectors either IN or IM in a prime-boost-boost vaccination regime (Fig. S1A). A cH5/3 DNA prime was given to all groups. Based on previous studies, a vector vaccination order of ChAdOx1 followed by MVA shows the strongest immune response induction, and will be consistently applied in this study. Four weeks post MVA boost, the mice were challenged with 5x 50% lethal doses (LD_50_) of X-31 (H3N2; HA and NA from A/Hong Kong/1/1968 in an A/Puerto Rico/8/1934 backbone), and weight loss and survival was observed over 14 days. Overall, we observe that mice vaccinated with a DNA prime, ChAdOx1 cH14/3, and MVA-cH15/3 intramuscularly showed reduced weight loss and were completely protected from an X-31 challenge, specifically in comparison to groups that received the corresponding recombinant proteins (Fig. S1B). A primary and secondary boost of ChAdOx1 cH14/3 followed by MVA-cH15/3 led to lower weight loss in mice, relative to a sequence of ChAdOx1 cH15/3 and MVA-cH14/3. Mice vaccinated intranasally with viral vectors or recombinant protein both were completely protected and lost minimal weight (Fig. S1C). Control viral vectors expressing GFP also conferred complete protection against lethal X-31 challenge, suggestive of a non-specific mucosal immune response induced by delivering viral vectors IN. Since this unspecific background makes challenge studies impossible, IM vaccination was finalized as the route of administration for future experiments to clearly delineate immune responses specific to the influenza virus antigens encoded by viral vectors.

### 3.2 Determination of the optimal influenza virus challenge setting

Prior to assessing the protective efficacy of bivalent viral vector vaccination (expressing cHAs and NP+M1) in mice, a dose titration of group 2 challenge viruses was carried out. To determine an optimal challenge dose, mice were IM vaccinated with viral vectors (VV), ChAdOx1 and MVA, expressing NP+M1 or GFP as a control (VV NP+M1 and VV Control), in a prime-boost regimen 3 weeks apart. This was not performed for H7N9 due to unavailability of sufficient GFP expressing vectors at the time. Six weeks post-boost, groups of mice were challenged with log incremental titers of A/Philippines/2/82 (H3N2), A/Shanghai/1/2013 (H7N9) and A/Jiangxi-Donghu/346/2013 (H10N8) (Fig. 1A). This was performed to identify a challenge dose that would induce significant weight loss in mice vaccinated with NP+M1-expressing vectors but would be lethal in animals vaccinated with vectors expressing GFP to ensure we could adequately monitor any combined protective efficacy post-vaccination with both cHA and NP+M1 (Fig. 1B-D). It also demonstrated the protective efficacy of vaccination with NP-M1 constructs alone, as the LD_50_ values in GFP vaccinated animals were clearly lower than the LD_50_ values in animals vaccinated with vectors expressing NP and M1. The corresponding Kaplan-Meier survival plots are shown (Fig. S2). We determined the optimal challenge doses as 10^2^ pfu/mouse for A/Philippines/2/82 (H3N2), 10^4^ pfu/mouse for A/Shanghai/1/2013 (H7N9), and 2 × 10^3^ pfu/mouse for A/Jiangxi-Donghu/346/2013 (H10N8). Using these challenge doses, the increase in protective efficacy with bivalent over monovalent viral vectors can be efficiently assessed with an abrogation of this weight loss.

### 3.3 cHA-NP+M1 vaccination protects mice against influenza virus challenge

To assess the breadth of protection in mice vaccinated with viral vectors expressing both cHA and NP+M1, we immunized mice using prime-boost/prime-boost-boost regimens (Fig. 2A). Mice in the VV cHA with cHA Boost group were vaccinated with monovalent viral vectors expressing only the chimeric HAs (as tested in Fig. S1) followed by a recombinant cHA protein boost to induce a strong antibody response against the HA stalk. VV NP+M1 group mice were vaccinated with viral vectors expressing the NP+M1 fusion protein. Groups labeled as VV cHA-NP+M1 Late and VV cHA-NP+M1 Early were vaccinated with viral vectors expressing NP+M1 and cHAs, with a difference in time of vaccination relative to viral challenge. This approach was taken to test if a longer interval between the last vaccination and challenge would negatively impact on the protective effect of the vaccine. The VV Control, Mock Control, and Naïve groups were vaccinated with GFP-expressing vectors, irrelevant recombinant protein with adjuvant or were completely naïve, respectively.

Following IN challenge with H3N2, VV cHA with cHA Boost, VV cHA-NP+M1 Late, and VV cHA-NP+M1 Early groups (all expressing cHAs) were completely protected from mortality (Fig. 2B). However, mice receiving cHA vaccines only or NP-M1 only (in VV cHA with cHA Boost and VV NP+M1, respectively) showed considerable weight loss with one mouse succumbing to infection in the VV NP+M1 group. This was expected from the challenge dose finding experiments and allowed for a window in which an improvement of combining the two approaches can be observed. Groups receiving combinations of viral vectors expressing NP+M1 and cHA outperformed other vaccinated groups showing minimal weight loss post challenge. The morbidity and mortality seen in the control groups (VV Control, Mock Control, and Naïve) were high, as expected.

The benefits of combining approaches were present but less pronounced for H10N8 challenged mice. Mice in the VV NP+M1 group underwent faster initial weight loss until day 3 post challenge than all other groups but started to regain weight by day 5 and were completely protected from mortality. However, these are qualitative observations and substantially larger group numbers would be required in order to determine statistical significance of these differences in weight loss dynamics and recovery rates. Vectored cHA vaccination (VV cHA with cHA Boost) protected most mice well from severe weight loss but one mouse succumbed to infection. Mice in the VV cHA-NP+M1 Early and Late groups showed minimal weight loss and were completely protected against H10N8 lethal challenge with the exception of one mouse in the VV cHA-NP+M1 Early group (Fig. 2C). Three mice survived in the viral vector GFP (VV Control) group, albeit with severe weight loss (24%) and late, incomplete recovery. All animals in the other control groups (Mock Control and Naïve) succumbed to infection.

**Fig. 3:**
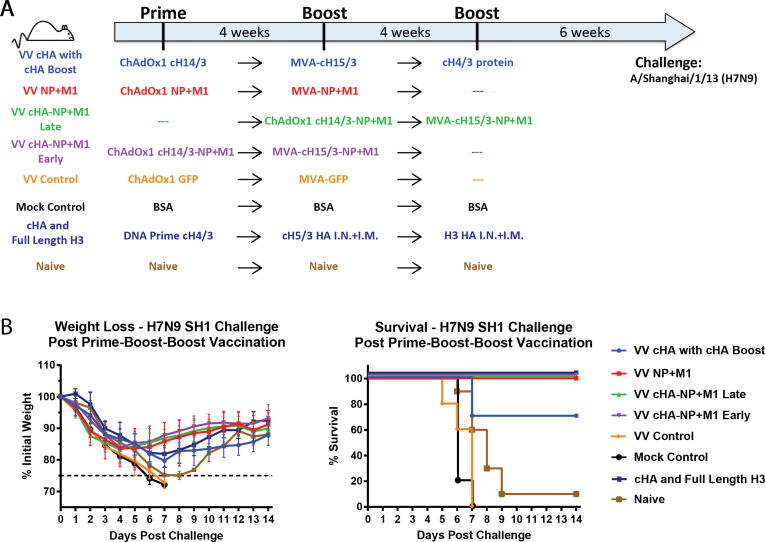
Assessing the protective effect of viral vectored vaccines against H7N9. (A) Mice were vaccinated intramuscularly as shown in a prime-boost or a prime-boost-boost regimen with four weeks between each vaccination. Groups VV cHA with cHA Boost, VV NP+M1, VV cHA-NP+M1 Early, VV cHA-NP+M1 Late, VV Control, Mock Control, cHA and Full Length H3 received vectored cHAs, vectored NP+M1, vectored cHA-NP+M1 (Early & Late), vectored GFP control, irrelevant protein vaccinations or cHA/H3 full length proteins (as previously published) respectively. Six weeks after the boost, mice were challenged IN with 2x10^3^ pfu/mouse A/Shanghai/1/13 (H7N9, PR8 reassortant). (B) Weight loss and Kaplan-Meier survival plots are shown over 14 days post challenge. Weight loss is shown as mean of the group with error bars representing SD. *n* = 5–10 mice/group. The figure represents pooled data from two separate experiments.

**Fig. 4:**
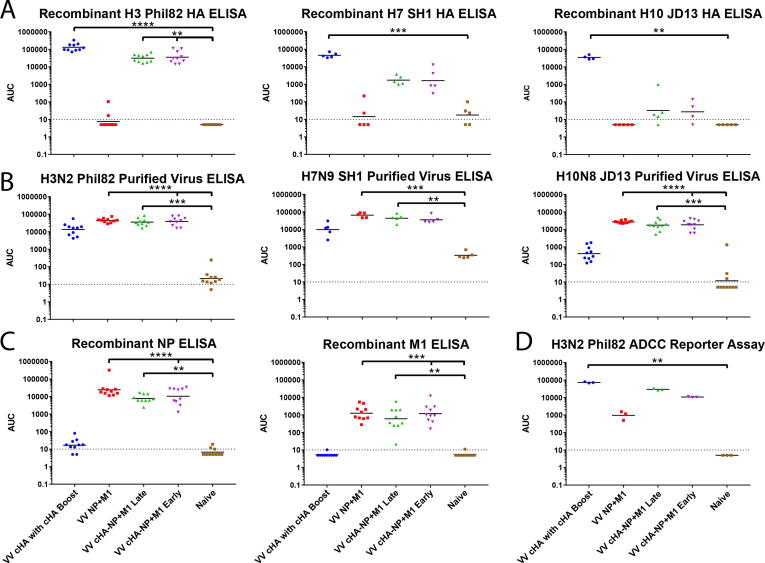
Characterizing antibody responses in pre-challenge sera of vaccinated mice. Six weeks after the boost vaccination, pre-challenge serum was collected from all groups of mice and IgG antibody responses against (A) recombinant group 2 HAs (matched to challenge strains), (B) purified group 2 influenza viruses (same as challenge strains) and (C) recombinant NP and M1 (from A/Puerto Rico/8/34) were assessed. (D) The sera were also evaluated in an *in vitro* ADCC reporter assay to assess activity of ADCC induction against MDCK cells infected with A/Philippines/82 (H3N2, PR8 reassortant). The data is represented as area under the curve calculated with a cut-off of average of blanks + 3 x SD of blanks. Statistical significance is denoted in comparison to naïve mice as determined by Kruskal-Wallis one-way ANOVA (*p < 0.05; **p < 0.01; ***p < 0.001; ****p < 0.0001).

The situation is more complex for the H7N9 challenge. Here, vectored cHA vaccination (VV cHA with cHA Boost) led to only 70% survival while all groups receiving NP+M1 or cHA+NP+M1 had 100% survival (Fig. 3B). There was no difference between these groups in terms of weight loss (all approx. 15%) suggesting that combining strategies did not add a benefit to protection afforded with vaccination against NP+M1 alone. Since cHA vaccination had provided 100% protection from H7N9 challenge with a slightly different vaccination regimen (cHA DNA followed by two recombinant protein vaccinations [11]), we also added a bridging group (‘cHA and Full Length H3′) to asses if the cHA vectored approach was inferior to the DNA-prime/protein boost approach used earlier [11]. The DNA-prime/protein boost animals recovered slightly faster than the cHA-vectored group and showed 100% survival (with the difference between the groups not being statistically significant). Of note, these results are pooled from two separate experiments with the VV cHA with cHA, VV cHA-NP+M1 Early and Naïve groups being present in both. Antibody titers in the shared groups were similar in both experiments (Fig. 4A and Suppl. Fig. 4).

Overall, vaccination with viral vectors expressing cHA-NP+M1 in a prime boost regimen provides complete protection upon challenge with three different group 2 influenza A viruses. Benefits of combining vaccine strategies were more obvious for H3N2 than for H10N8 and were not observed for H7N9.

### 3.4 Characterization of the antibody response induced upon vaccination

To assess the humoral immune response to vaccination we performed ELISAs with pre-challenge serum (from mice vaccinated as shown in Fig. 2A), against a panel of group 2 HA recombinant proteins (Fig. 4A). VV cHA with cHA Boost, VV cHA-NP+M1 Late, and VV cHA-NP+M1 Early groups vaccinated with cHA expressing viral vectors showed high levels of H3-reactive antibodies post-vaccination. Mice in VV cHA with cHA Boost group were exposed to the H3 stalk three times including a recombinant protein boost, and showed the highest reactivity to H3. ELISA titers of animals that experienced cHAs twice (VV cHA-NP+M1 Late and Early) were four-fold lower. Reactivity was confirmed to be to the stalk and not to the tetramerization domain and hexahistidine tag using a recombinant HA protein that expressed a different trimerization domain and purification tag than the HA used for the protein boost (Fig. S3).

Antibody titers against other group 2 influenza HAs such as H7 and H10 HAs showed that a broadly cross-reactive response can be obtained through the vectored cHA vaccination strategy (cH14/3, cH15/3, cH4/3). However, we found that a robust cross-reactive response required three immunizations with cHA antigen as antibodies induced in VV cHA-NP+M1 Late and Early groups were not as cross-reactive as the antibodies induced in the VV cHA with cHA Boost vaccination regimen. Assessment of antibody responses against purified A/Philippines/2/82 (H3N2, X-79), A/Shanghai/1/2013 (H7N9, PR8 reassortant) and A/Jiangxi-Donghu/346/2013 (H10N8, PR8 reassortant) viruses showed strong anti-influenza virus antibody responses in all non-control vaccination groups (Fig. 4B). Interestingly, we also found strong reactivity in sera of VV NP+M1 animals, despite a lack of anti-HA antibodies. To investigate if this reactivity in the VV NP+M1 vaccination group could be mediated by antibodies to the internal proteins of the virus, we measured antibody responses to recombinant NP and M1 and found reactivity in groups that received vectors expressing the NP+M1 fusion construct (Fig. 4C).

To test whether the induced antibodies can induce Fc effector functions such as antibody-dependent cellular cytotoxicity, pooled sera were assayed in an *in vitro* ADCC reporter assay. Robust induction of ADCC was detected in sera from groups VV cHA with cHA Boost, VV cHA-NP+M1 Late and Early when tested against MDCK cells that were infected with A/Philippines/2/82 (H3N2, X-79) (Fig. 4D). The degree of ADCC induction was markedly lower for the VV NP+M1 group, indicating that the anti-HA stalk antibodies predominantly mediate ADCC activity in these pooled sera and that the contribution of anti-NP and anti-M1 antibodies is relatively small.

### 3.5 Vaccination with viral vectors expressing NP+M1 induces strong IFNγ CD8+ T cell responses in mice

**Fig. 5:**
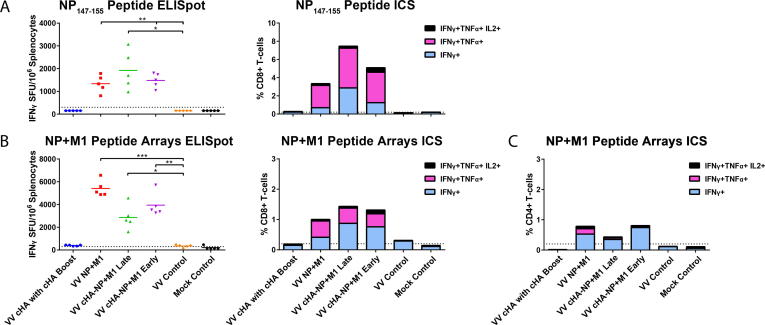
Influenza virus-specific IFNγ responses induced by vaccinating mice with NP+M1 expressing viral vectors. Three weeks after the final boost, spleens were removed from mice and splenocytes were assessed for IFNγ production (via ELISpots) and influenza virus specific CD8+ T cells (via intracellular cytokine staining (ICS) assay) following stimulation with the (A) NP_147-155_ immunodominant epitope, and (B) peptide array pools spanning NP+M1 fusion protein. (C) CD4+ T cell responses were studied in an ICS assay following stimulation by peptide array pools spanning the NP+M1 fusion protein. The level of polyfunctionality of the T cells is also denoted in context of IFNγ+, TNFα, IL-2 secretion. Results are expressed as individual data points with mean values denoted by horizontal lines for ELISpots and as % of antigen specific CD8+/CD4+ T cells in pooled samples for ICS. Statistical significance is denoted in comparison to naïve mice as determined by one-way ANOVA (*p < 0.05; **p < 0.01; ***p < 0.001; ****p < 0.0001). n = 5 mice per group.

Given that the strategy to express NP and M1 by viral vectors is primarily aimed at inducing cellular immune responses, we also assessed the induction of influenza virus specific CD8+ T-cells. We harvested splenocytes three weeks after the final boost and stimulated these cells with the BALB/c restricted immunodominant NP_147-155_ epitope. Using the ELISpot assay, we found increased numbers of influenza virus-specific IFNγ expressing cells following stimulation with the NP_147-155_ immunodominant peptide (Fig. 5A) in mice that received monovalent or bivalent viral vectors expressing the NP+M1 fusion protein (VV NP+M1, VV cHA-NP+M1 Late, and VV cHA-NP+M1 Early). These results were confirmed by ICS using stimulation with the same peptide. The majority of detected cells in VV NP+M1, VV cHA-NP+M1 Late, and VV cHA-NP+M1 Early groups were polyfunctional and expressed both IFNγ and TNFα, fewer cells expressed only IFNγ and a small percentage expressed IFNγ, TNFα and IL-2. Responses were strongest in the VV cHA-NP+M1 Late group followed by VV NP+M1 and VV cHA-NP+M1 Early groups, which were similar. Increases were also observed when splenocytes were stimulated with pooled peptides spanning the entire NP+M1 fusion polypeptide and analyzed in IFNγ ELISpot and ICS assays (Fig. 5B). In this case, the VV NP+M1 group showed the strongest signal in ELISpot while VV NP+M1, VV cHA-NP+M1 Late, and VV cHA-NP+M1 Early groups were similar in ICS. No reactivity in any of the assays was detected for the VV cHA with cHA Boost or the control groups. For CD4+T-cells, the responses observed in the VV NP+M1 and VV cHA-NP+M1 Early groups were similar. The overall induction of CD4+T-cells was low and cells expressing only IFNγ were the predominant population (Fig. 5C). To summarize, these results suggest that vectored vaccines can simultaneously induce cellular and humoral immune responses to multiple influenza virus antigens, which may lead to broad, long-lasting protection against influenza viruses.

## 4 Discussion

Universal influenza virus vaccines typically target conserved epitopes of the virus. Here we combined a strategy to induce antibodies to the stalk domain using sequential vaccination with cHAs with a vaccine strategy that concurrently induces cellular immunity to conserved epitopes on NP and M1. This was achieved by co-expression of an NP+M1 fusion protein and different cHAs using ChAdOx1 and MVA replication deficient vectors. Combining these two strategies was successful and we did not measure any cellular immune interference toward NP+M1 due to expression of a second antigen which has previously been observed in viral vectored vaccines [42]. Although one vector might be sufficient, multiple studies using the ChAdOx1 – MVA prime boost regimen for a variety of viral glycoproteins have shown to be superior to a singular vector prime. This specifically manifests in terms of the cellular responses, and would likely also benefit the antibody induction against glycoproteins. Our data suggests that T cell responses in mice vaccinated with the bivalent viral vectors induce robust cellular immune responses. Importantly, as demonstrated by very similar protection afforded by the two different VV cHA-NP+M1 groups (Early and Late), the induced T cell responses by these vectors seem also durable, at least in the observed time frame (6 weeks post boost versus 10 weeks post boost). This is an important finding since a universal influenza virus vaccine that does not induce durable protection is of limited use although it is unclear if this finding in the mouse model translates to humans.

Immune responses to the stalk domain are typically group-specific and either target group 1 (H1, H2, H5, H6, H8, H9, H11, H12, H13, H16, H17 and H18) or group 2 (H3, H4, H7, H10, H14, and H15), with exceptions on the monoclonal level [1]. Significant work has been performed for group 1 – based vaccines [7], [8], [10], [12], [13], [15], [16], [17], [43], [44], [45] but development of group 2 vaccines is lagging behind [11], [14], [46]. However, H3N2 is currently the subtype that causes most morbidity and mortality in humans and both H7N9 and H10N8 have caused zoonotic infections. Furthermore, levels of antibodies that target the group 2 HA stalk are much lower in the human population than antibodies that target the group 1 HA stalk [47], [48]. Therefore, we decided to test group 2 cHAs as immunogens and group 2 HA expressing viruses, including H3N2, H7N9 and H10N8, for testing vaccine efficacy in the mouse model. Importantly, these viruses only carry the HA and neuraminidase of the respective wild type viruses, their other six genomic segments are derived from H1N1 strain A/PR/8/34. This makes them pathogenic for mice (A/PR/8/34 is mouse adapted). Of note, the NP and M1 expressed by the viral vectors tested are derived from recent H3N2 isolates and therefore are heterologous to the NP and M1 proteins of the challenge viruses. Challenge doses were initially titrated, selecting a dose that induced significant weight loss in the NP+M1 vector-vaccinated animals. For H3N2 challenge both the cHA only and the NP+M1 only vaccination provided protection but combining the approach clearly led to an increase in protection as evidenced by minimal weight loss (morbidity) and complete survival. The benefits could also be seen, although to a lesser degree, in H10N8 challenged animals and were absent in H7N9 challenged animals. An explanation for this could be that vaccinating twice with two different cHAs induced high antibody titers against the H3 stalk but cross-reactivity to H7 and H10 was weak. A third boost was needed to induce stronger cross-reactivity, potentially due to additional affinity maturation. It is unclear if this is an artifact of the mouse model, but three vaccinations were also required in past studies to induce high levels of cross-reactive anti-stalk responses [7], [8], [10], [11], [12], [13], [14], [15], [16], [17]. Humans have low levels of pre-existing anti-group 2 stalk immunity and are therefore primed already. Giving two vectored vaccines would therefore resemble a prime-boost-boost regimen in a naïve animal model and would likely boost cross-reactive antibodies very efficiently. An alternative possibility is, that the cross-reactivity was driven by the combination of viral vectored followed by recombinant protein vaccine. We also found high reactivity of antibodies to recombinant NP and M1 protein in all animals vaccinated with viral vectors expressing the NP+M1 fusion protein. These antibodies, due to the internal nature of their targets, likely have difficulties to interact with NP and M1 in intact virions or infected cells. However, it has recently been shown that human antibodies to these two proteins can activate effector cells and high anti-NP antibody titers have also been demonstrated to be protective in a mouse model [49], [50], [51]. When measured in an H3N2 ADCC reporter assay, sera from groups vaccinated with cHA expressing vectors showed high activity – likely the main protective mechanism for stalk antibodies (neutralization was not observed in this study). However, the activity of sera from mice vaccinated only with NP+M1 in this assay was low. The relevance of anti-NP and anti-M1 antibodies, in addition to strong CD8+ T-cell responses against the same proteins, remains therefore unclear. In addition, T-cells targeting conserved epitopes of the HA [52] might also contribute to protection but have not been evaluated in this study. It has been demonstrated that pre-existing CD4+ T-cell immunity toward HA, generated by seasonal influenza viruses, can augment the humoral response toward both homologous and heterologous HA and will be important to consider when studying our vaccine in a influenza virus-exposed population [53], [54], [55], [56].

In summary, our data shows that vaccination with cHA, NP, and M1 delivered by ChAdOx1 and MVA viral-vectored vaccines induces potent T and B cell responses which can confer broad protection in a rigorous challenge model. Overall, these results improve our understanding of vaccination platforms capable of harnessing cellular and humoral immunity with the ultimate goal of designing a universal influenza virus vaccine.
