# Supplementary material for: Vaccination with viral vectors expressing NP, M1 and chimeric hemagglutinin induces broad protection against influenza virus challenge in mice
Source: Vaccine. 2019 Sep 3;37(37):5567–77. doi: 10.1016/j.vaccine.2019.07.095 (PMC6717082; doi:10.1016/j.vaccine.2019.07.095)
Supplement: Supplementary data 1 [file mmc1.docx]

**Supplementary methods**

To assess the optimal route of vaccination and construct sequence, mice were divided into 7 experimental groups (**Fig. S1A)**. All groups received a cH5/3 DNA prime. For this, 65µg of pCAGGS mammalian expression vector encoding for cH5/3 were injected intramuscularly with an accompanying electrical stimulus following injection (TriGrid delivery, Ichor Medical Systems). The following boosts for group A to F were carried out intranasaly and intramuscularly to determine the optimal route of administration for the vaccines. Four weeks post prime, group A, group C, and group E received 10^8^ IU/50µL in PBS of ChAdOx1 cH14/3, ChAdOx1, cH15/3, and ChAdOx1 GFP respectively. Correspondingly, groups B and D received 5µg recombinant cH14/3 and cH15/3 HA adjuvanted with 5µg polyI:C respectively. Group F received an irrelevant protein control, 5µg of BSA adjuvanted with 5µg polyI:C. Four weeks post boost, group A received 10^6^ IU/50µL of PBS of MVA-cH15/3 while group C received MVA-cH14/3. Group E received the same dose of a control MVA-GFP vector as a secondary boost to the initial ChAdOx1 GFP boost. Group B and group D received 5µg of cH15/3 or cH14/3 recombinant HA adjuvanted with 5µg polyI:C as the secondary boost respectively. Group F was boosted again with 5µg BSA and 5µg polyI:C. Mice in group G were maintained as unvaccinated naïve controls. 4 weeks post-secondary boost, pre-challenge sera was collected from these mice and these mice were challenged with a 5xLD_50_ dose of X-31 (H3N2). Weight loss and survival was monitored for 14 days with a 25% weight loss cut-off set as a humane end point, above which mice were sacrificed.


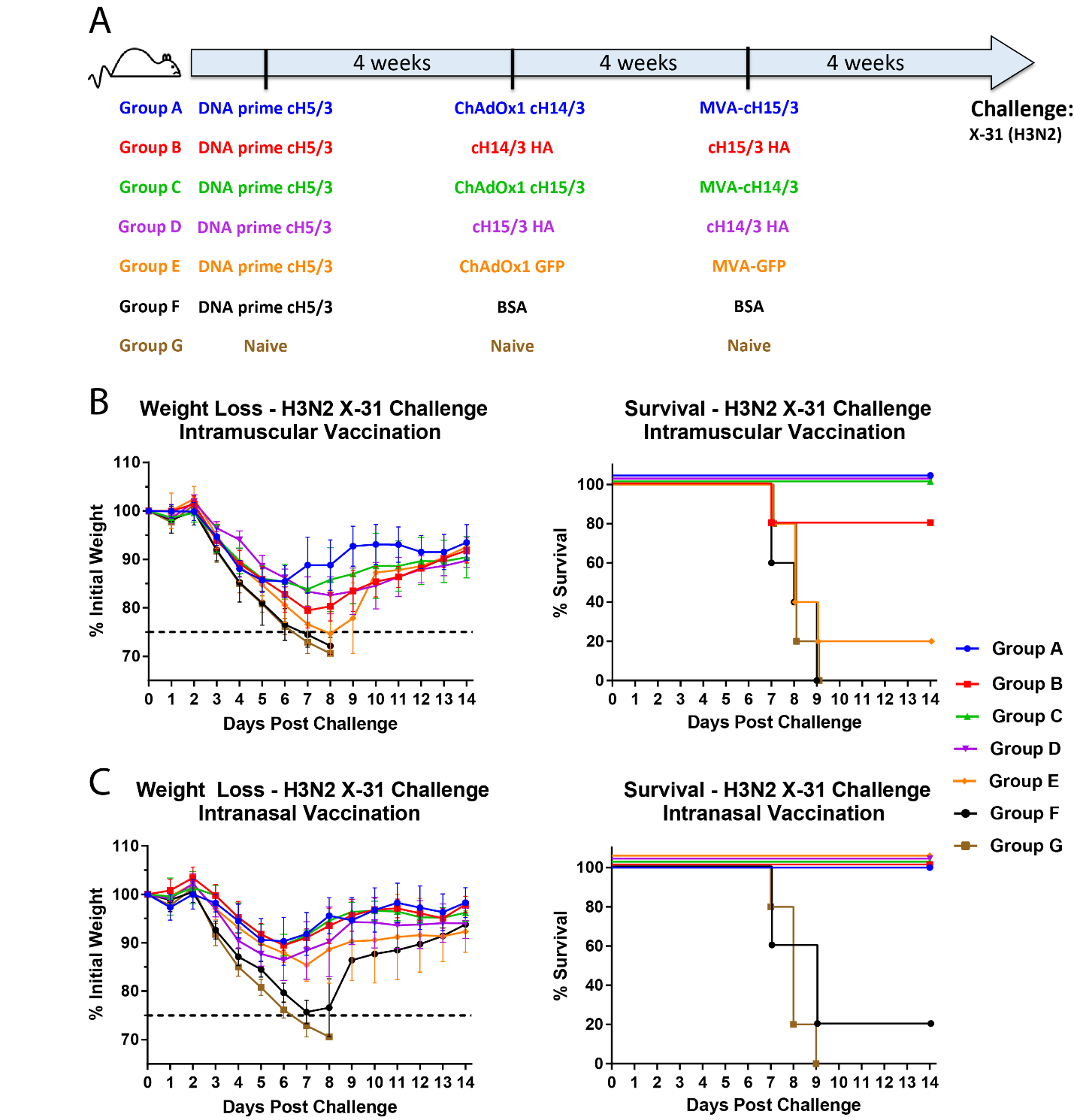


**Supplementary Figure 1. Protective effects of a ChAdOx1 cH15/3 and MVA-cH14/3 prime-boost vaccination regime via the IM or IN route. (A)** Mice were vaccinated as shown in a prime-boost-boost vaccination regimen in both an IM and IN route. They were challenged with 5 x LD_50_ A/X31, and monitored over 14 days. **(B) & (C)** Weight loss and Kaplan-Meier survival plots are shown for vaccinated mice via the IM and IN route, respectively. The dashed line in the weight loss graphs represents a 75% of initial weight, a humane end-point below which the mice are sacrificed. Weight loss is shown as mean of the group with error bars representing SD. *n* = 5 mice/group.


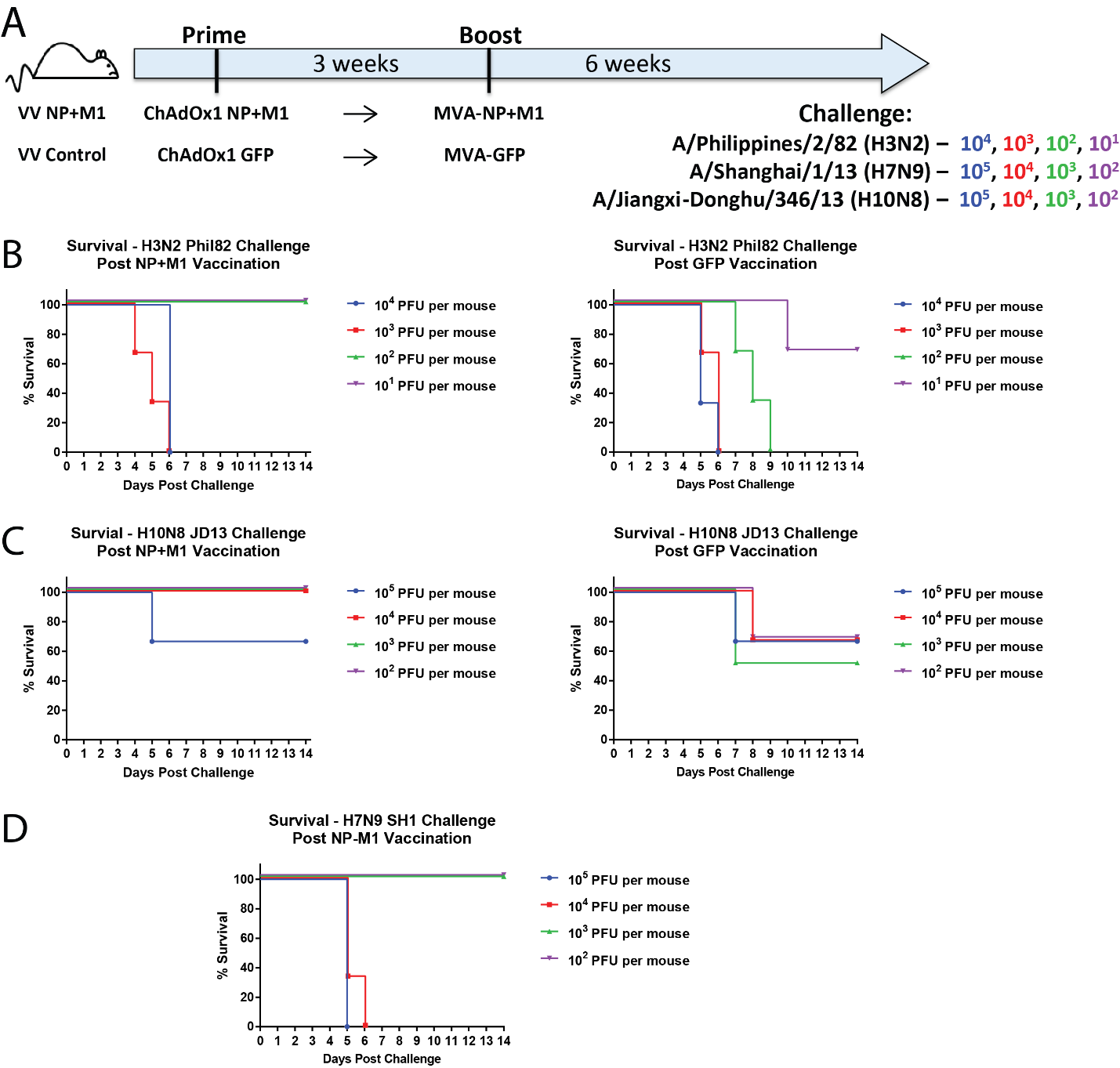


**Supplementary Figure 2.** Kaplan-Meier survival plots are shown for the A/Philippines/2/1982 (H3N2, X-79), A/Jiangxi-Donghu/346/2013 (H10N8, PR8 reassortant) and A/Shanghai/1/2013 (H7N9, PR8 re-assortant) challenge, in correspondence with the scheme described in **Fig 1.**


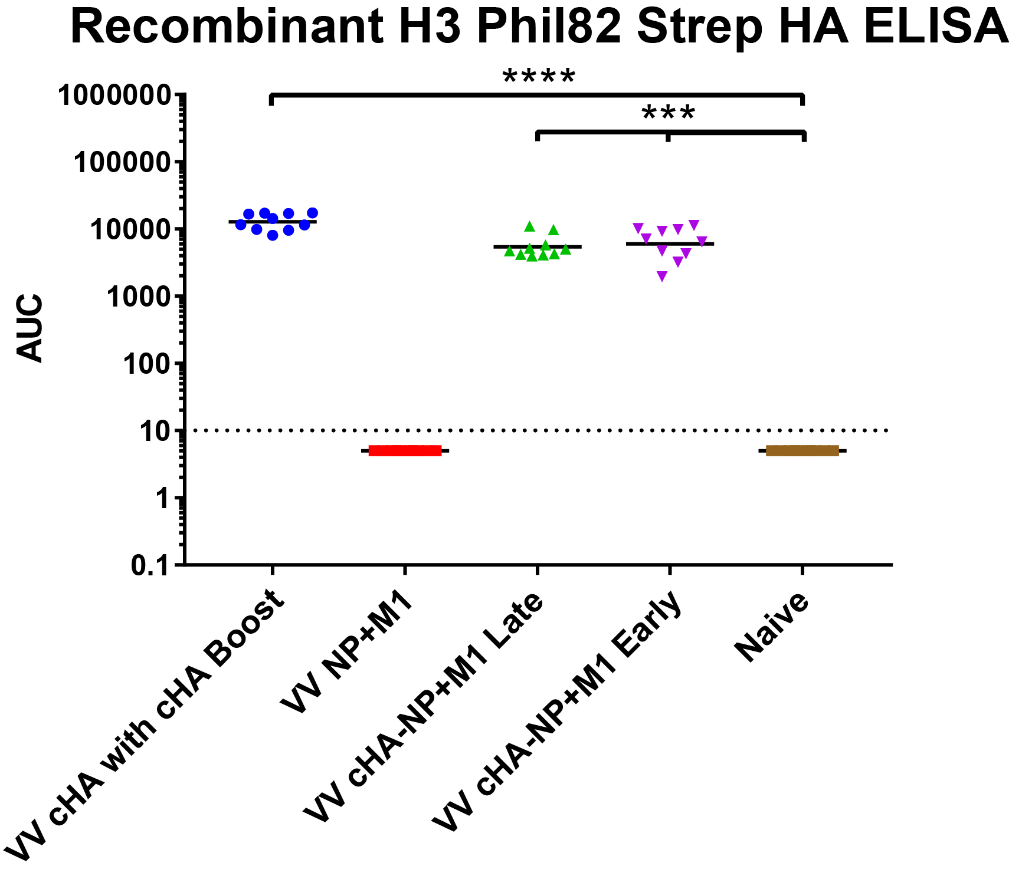


**Supplementary Figure 3. Characterizing the antibody response specifically against the H3 hemagglutinin in pre-challenge sera of vaccinated mice.** Pre-challenge sera were collected from vaccinated mice and the IgG response against a streptavidin tagged H3 hemagglutinin was tested in an ELISA. The data is shown as area under the curve with a cut-off of average of blanks + 3 x SD of blanks. Statistical significance is denoted relative to naïve mice as determined by Kruskal Wallis one-way ANOVA (*p < 0.05; **p<0.01; ***p<0.001; ****p<0.0001)


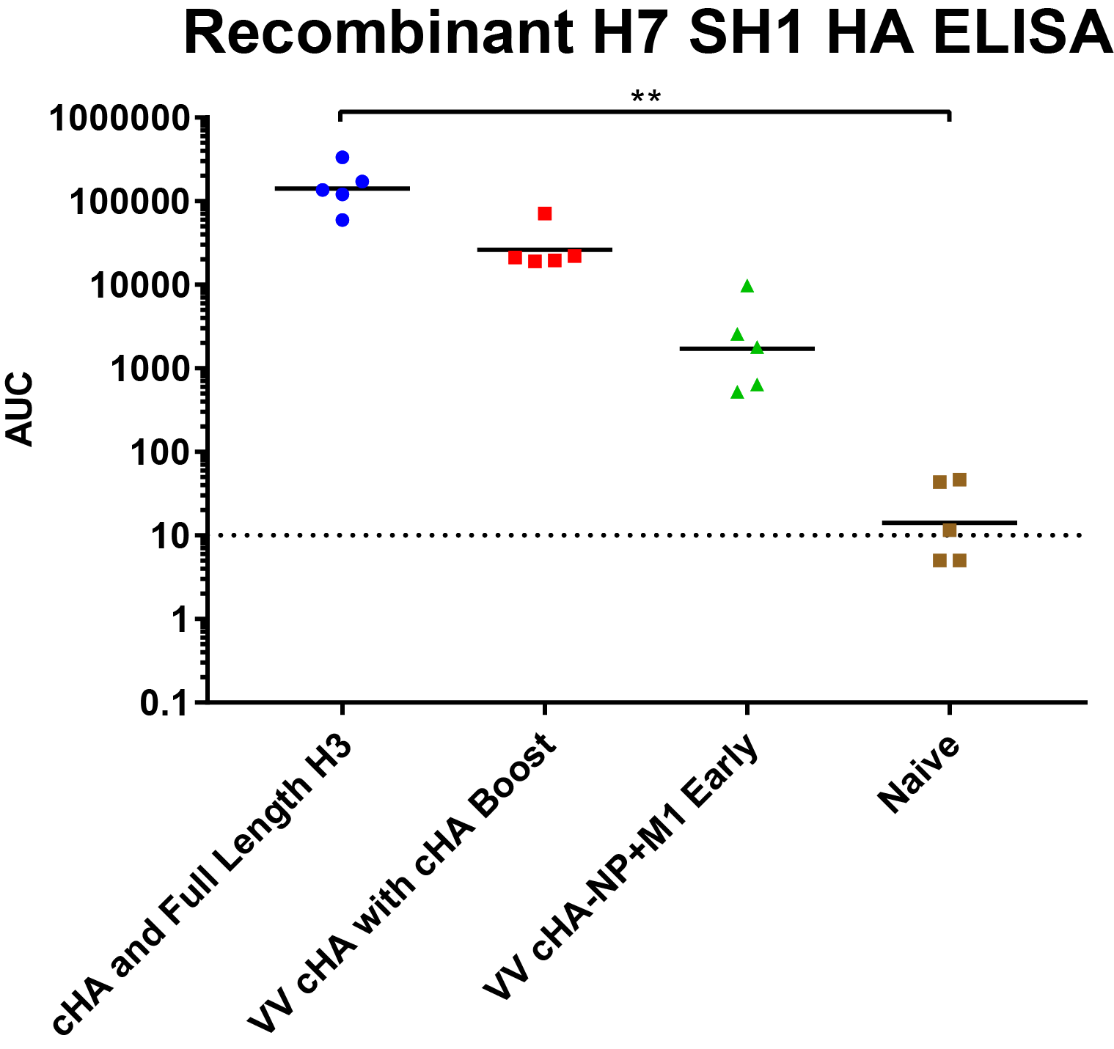


**Supplementary Figure 4. Characterizing antibody responses in pre-challenge sera of vaccinated mice from the second part of the H7N9 challenge experiment which includes the ‘cHA and Full Length H3’ bridging group.** Pre-challenge sera was collected from vaccinated mice as shown in **Fig. 3** prior to a challenge with A/Shanghai/1/13 (H7N9). The IgG response was tested against a recombinant H7 protein matched to the challenge strain. The data is shown as area under the curve calculated with the average + 3 x SD of blanks as the cutoff. Statistical significance is denoted in comparison to naïve mice as determined by one-way ANOVA (*p < 0.05; **p < 0.01; ***p < 0.001; ****p < 0.0001). n = 5 mice per group.
